# A Personal Decision Support System for Heart Failure Management (HeartMan): study protocol of the HeartMan randomized controlled trial

**DOI:** 10.1186/s12872-018-0921-2

**Published:** 2018-09-27

**Authors:** Anneleen Baert, Els Clays, Larissa Bolliger, Delphine De Smedt, Mitja Lustrek, Aljoša Vodopija, Marko Bohanec, Paolo Emilio Puddu, Maria Costanza Ciancarelli, Michele Schiariti, Jan Derboven, Gennaro Tartarisco, Sofie Pardaens, on behalf of the HeartMan consortium

**Affiliations:** 1 Department of Public Health, Ghent University, University Hospital Ghent, entrance 42 (4K3) Corneel Heymanslaan 10, 9000 Ghent, Belgium; 2 Department of Intelligent Systems, Jožef Stefan Institute, Ljubljana, Slovenia; 3 Department of Knowledge Technologies, Jožef Stefan Institute, Ljubljana, Slovenia; 4 Department of Cardiovascular Sciences, Sapienza University of Rome, Rome, Italy; 5 Mintlab, KU Leuven, Leuven, Belgium; 6 Italian National Research Council (CNR) – Institute of Applied Science and Intelligent System (IASI), Messina Unit, Messina, Italy; 7 Onze-Lieve-Vrouw Hospital Aalst, Cardiovascular Center, Aalst, Belgium

**Keywords:** mHealth, Disease management, Heart failure, Health-related quality of life, Decision support system

## Abstract

**Background:**

Heart failure (HF) is a highly prevalent chronic disease, for which there is no cure available. Therefore, improving disease management is crucial, with mobile health (mHealth) being a promising technology. The aim of the HeartMan study is to evaluate the effect of a personal mHealth system on top of standard care on disease management and health-related quality of life (HRQoL) in HF.

**Methods:**

HeartMan is a randomized controlled 1:2 (control:intervention) proof-of-concept trial, which will enrol 120 stable ambulatory HF patients with reduced ejection fraction across two European countries. Participants in the intervention group are equipped with a multi-monitoring health platform with the HeartMan wristband sensor as the main component. HeartMan provides guidance through a decision support system on four domains of disease management (exercise, nutrition, medication adherence and mental support), adapted to the patient’s medical and psychological profile. The primary endpoint of the study is improvement in self-care and HRQoL after a six-months intervention. Secondary endpoints are the effects of HeartMan on: behavioural outcomes, illness perception, clinical outcomes and mental state.

**Discussion:**

HeartMan is technologically the most innovative HF self-management support system to date. This trial will provide evidence whether modern mHealth technology, when used to its full extent, can improve HRQoL in HF.

**Trial registration:**

This trial has been registered on https://clinicaltrials.gov/ct2/show/NCT03497871, on April 13 2018 with registration number NCT03497871.

## Background

About 1–2% of people in the western world suffer from heart failure (HF) [1]. Although treatment improvements have decreased the number of hospitalizations and deaths due to HF, the burden remains high with half of the HF patients being expected to die within five years after diagnosis, and HF being the most frequent cause of hospitalization in people aged over 65 [2].

Since there is presently no cure available, a better management of HF is crucial. Proper disease management may relieve symptoms, prevent hospitalization or improve survival, but may also affect the patient’s health-related quality of life (HRQoL). The European Society of Cardiology (ESC) Task Force produced guidelines for the diagnosis and management of HF, intended to be used by clinical practitioners [3]. However, previous studies consistently show an insufficient uptake of these guidelines in clinical practice [4]. Of particular concern is the poor implementation of exercise guidelines [5]: participation rates of HF patients in cardiac rehabilitation are generally below 20% in Europe [6]. This calls for action to develop strategies to give appropriate and effective personalized lifestyle advice to HF patients.

A promising technology for improving disease management in HF may be mobile health (mHealth), which encompasses the use of mobile devices as a support to clinical practice. mHealth has already been implemented in HF patients to provide regular follow-up and physiological monitoring, to ensure safety and to detect complications [7]. In addition, it may be a tool to deliver education and support patients regarding self-monitoring and self-management. However, evidence on the effectiveness of this approach in HF patients has been diverse. A recent meta-analysis suggests clinical benefits on all-cause mortality and heart failure related hospitalisations [8], but large clinical trials did not show any effect on readmission or death [9, 10]. However, the BEAT-HF trial found despite the absence of reduced rehospitalization or mortality, an improvement in HRQoL [11].

HRQoL and perceived health status, both patient-reported outcomes (PROs), have increasingly been recognized as outcomes of interest in HF and coronary patients [12, 13]. PROs are not surrogates for harder endpoints such as mortality, but rather represent independent outcomes [14]. Nevertheless, some studies report that HRQoL and health status may be predictive of clinical events in HF [15, 16], suggesting a relation between both types of outcome. These findings indicate that patients’ perceptions of worsening symptoms might carry vital prognostic information, and should be implemented in trials evaluating disease management.

An aspect that has been largely ignored in mHealth trials is the psychological aspect that is necessary to start changing behaviour and to cope with HF symptoms. Psychological interventions such as cognitive behavioural therapy and mindfulness exercises have already been shown to be successful in changing lifestyle behaviour [17, 18] and to significantly reduce anxiety, depression and clinical symptoms which are common in HF patients [19]. Therefore, implementing psychological interventions in mHealth technology may offer an added value.

This paper presents the study protocol of HeartMan (*Personal Decision Support System For Heart Failure Management)* which aims to develop a personal health system to improve disease management and HRQoL in HF. In this system, patients’ monitoring is focused on their physical condition and psychological state. This data is integrated into a decision support system (DSS), which is an information system supporting complex decision making processes. In HeartMan, the DSS suggests the most appropriate intervention (including exercise, nutrition, medication and mental support) to modify and manage the patient’s lifestyle, adapted to his psychological profile in order to increase adherence to the medical advice.

## Study design

### Design

HeartMan is a randomized controlled 1:2 (control:intervention) proof-of-concept trial, being conducted across two countries (Belgium and Italy) to compare standard care in HF with the addition of a personal mHealth system on top of standard care. In each country 60 patients are enrolled for a six-month period. Hence 40 patients in total are included in the control group and 80 in the intervention group.

### Study objectives and outcome measures

The overall objective of HeartMan is to improve disease management, resulting in an improvement in HRQoL after a 6 months intervention period.

Secondary objectives are the effects of HeartMan on behavioural outcomes, illness perception and clinical outcomes which may impact disease management and HRQoL. Additional secondary aims encompass the effects of psychological interventions within HeartMan and a user-friendly design of the HeartMan system.

Primary and secondary outcome measures, which are assessed in the intervention and control group, are listed below. These measures are collected at start and end of the study, unless otherwise stated.

#### Primary objective

- The primary endpoint is the self-reported improvement in self-care and HRQoL, measured by the Self-care of Heart Failure Index [20] and Minnesota Living with Heart Failure Questionnaire [21]

#### Secondary objectives

- Effect of HeartMan on behavioural outcomes, which are:
  - Adherence to dietary recommendations, measured by a self-composed questionnaire on nutritional knowledge and eating behaviour
  - Activity behaviour, daily measured by the number of calories (via an accelerometer in the HeartMan wristband sensor)
  - Medication adherence by questioning the patient on their medication intake on a weekly basis
  - Sexual activity, assessed with the Sexual Adjustment Scale [22] and Needs for Sexual Counselling Scale in chronic HF [23]
- Effect of HeartMan on illness perception, evaluated with the Brief Illness Perception Questionnaire [24]
- Effect of HeartMan on exercise tolerance, measured by:
  - Change in resting heart rate and heart rate during exercise
  - Distance obtained at the six–minute walking test (6MWT) [25]
- Effect of psychological interventions in HeartMan – cognitive behavioural therapy and mindfulness exercises – on anxiety and depressive feelings, measured with State Trait Anxiety Inventory [26] and the Beck Depression Inventory II [27]
- Evaluation of the user experience of HeartMan to assess the expectations towards the system and the patient’s experiences (only measured in the intervention group), evaluated with the Unified Theory of Acceptance and Use of Technology questionnaire, [28] adapted to the objectives of the HeartMan system and to the population of elderly users [29]

##### Sample size calculation

Sample size calculations were based on primary outcome data from the CHIRON project [13], showing that 90 patients are needed to show a difference of 5.8 beats per min in average daily awake heart rate– as a fundamental parameter correlating with HRQoL – with 90% power between the two groups. In order to account for possible drop-outs, the trial is performed enrolling 120 patients – 60 in each of the two participating countries – based on a 1:2 (control:intervention) randomization protocol.

##### Study population

In order to be eligible for inclusion the following criteria were used:

- Willing and able to make use of a smartphone and to give informed consent for participation in the study
- Adults ≥18 years old
- Ischemic or non-ischemic HF disease
- Functional New York Heart Association (NYHA) class 2–3
- Reduced left ventricular ejection fraction ≤40%
- Ambulatory HF patients in stable condition: at least one hospitalization due to HF, but no hospitalization during the month before start of the trial and no planned surgery
- Good cognitive function, if cognitive impairment is suspected, the patient will be evaluated with Mini Mental State Examination (MMSE)
- Sufficient knowledge of the native language (Dutch in Belgium, Italian in Italy)

HF patients who fulfil the above mentioned criteria are excluded if:

- They suffer from a concomitant end-stage chronic kidney disease necessitating haemodialysis
- They are already participating in a disease management program influencing the HeartMan intervention

##### Investigation procedure

**Fig. 1:**
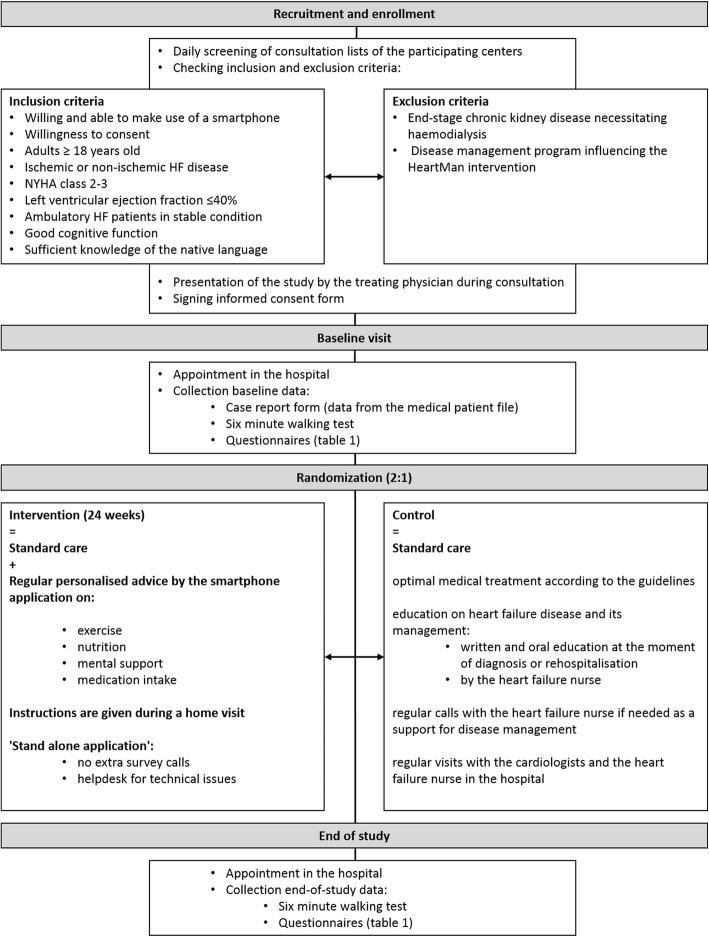
Study flow chart

A general overview of this study is presented in Fig. 1 and described in detail in following paragraphs.

##### Recruitment and enrolment

Patient recruitment is organized across three Belgian and one Italian hospital, representing one geographic area in each country. The participating hospitals in Belgium include one university hospital (University Hospital Ghent) and two local hospitals (AZ Maria Middelares Ghent and OLV Hospital Aalst). In Italy, one local hospital (Rieti General Hospital) as well as the local health authority (ASL Rieti) with their general physicians is involved.

The target population screened for participation are stable ambulatory HF patients who visit their treating cardiologist on a regular basis. At the time of consultation, the physician or HF nurse identifies eligible patients, briefly presents the study and asks about their interest in participation. If the patient shows interest, he is asked to come back for a scheduled appointment. Treating cardiologists and general physicians (for Italy) may also directly contact an eligible candidate, if the patient has no planned routine consultation in the immediate future.

##### Baseline visit

After patients have signed the informed consent form stating their willingness to be randomized, a baseline assessment is planned in the hospital maximum 20 weeks prior to the start of the trial. During this visit, all patients perform a 6MWT, which is in line with the recommendations of the Heart Failure Association of the ESC [5] to evaluate one’s exercise capacity in case cardiopulmonary exercise testing (CPET) is not available.

In addition, every participant receives a self-administered questionnaire package which is filled in during the baseline assessment. These questionnaires have previously been described in the study outcome measures section and details are listed in Table 1*.*

**Table 1:** Overview of the questionnaire package

Questionnaire	Description	Number of items	Subscales
Self – care of Heart Failure Index [20]	Self-care	22	Self-care behavior, self-care management, self-care confidence, and symptom perception
Minnesota Living with Heart Failure Questionnaire [21]	Health – related quality of life	21	Physical, socio-economic, and emotional/psychological aspects
Brief Illness Perception Questionnaire [24]	Illness perception	9	Cognitive representation, emotional representation, illness comprehensibility, and perceived cause of illness
Sexual Adjustment Scale [22] (subscale of the Psychosocial Adjustment Scale)	Sexual activity	6	Relationship and sexuality
Needs for Sexual Counseling Scale in Chronic Heart Failure [23]	Sexual activity	21	Symptoms, medication and information, relaxation, relationship, and psychological factors
State Trait Anxiety Inventory [26]	Anxiety	40	State anxiety scale (s-anxiety) and trait anxiety scale (t-anxiety)
Beck Depression Inventory II [27]	Depression	21	Depression and anxiety
Unified theory of acceptance and use of technology questionnaire [28]	User expectations and user experience	37	/
Self – composed questionnaire on nutritional behavior	Nutritional behavior	24	Drinking behavior and eating behavior
Self – composed questionnaire on nutritional knowledge	Nutritional knowledge	12 (14: patients with diabetes)	Knowledge about heart failure (and diabetes) nutrition

Following this assessment, patients are randomized to the intervention or control group according to a sealed randomization scheme with two balanced series of 60 envelopes (one for each country), each containing a random series of 20 control and 40 experimental numbers. In order to handle potential early drop-out of patients, a separate series of 24 sealed and balanced envelopes (12 for each country) is prepared.

Parallel to the baseline assessment, the following parameters are retrieved from the medical patient file as an additional base for providing personalized lifestyle advice: demographic and clinical characteristics, HF-related characteristics and laboratory parameters, risk factors, comorbidities, medication use and exercise capacity.

##### Intervention

Instructions concerning the HeartMan intervention tool are given to the intervention group during a home visit.

### Intervention equipment

Participants randomized to the intervention group are equipped with a multi-monitoring health platform able to monitor, process and fuse physio-psychological and behavioural data. The main component of the trial equipment is the HeartMan wristband sensor developed by BITTIUM (Oulo, Finland). This is an ambulatory recorder and transmitter for heart rate, heart rate variability, galvanic skin response, skin temperature, respiration rate and motion. This wristband has Bluetooth communication, recording function, display, haptic interface and the ability to synchronize with other external devices. Apart from the HeartMan wristband sensor, the equipment consists of registered and commercially available devices including a digital bathroom scale (ADE, Model Silje BE1303), upper arm blood pressure monitor (A&D Medical, Model Number UA-611) and a pill box organizer (PuTwo, 7-Day AM/PM Night Reminder Medi-Planner). Furthermore, a smartphone (Nokia 6 TA_1021) with HeartMan app installed is provided for the duration of the trial.

### Intervention components for the patient

Information coming from the trial equipment, baseline visit and medical patient file are integrated into the HeartMan DSS. The major component is personalized lifestyle advice on nutrition and exercise. The second intervention modality includes general notifications for medication intake with possibility to track the weekly consumption. The third component encompasses cognitive behavioural therapy and mindfulness exercises, which are offered for treating anxiety and depressive symptoms and to improve adherence. The fourth modality are reminders for physician’s appointments, which can additionally be activated upon the patient’s request. The fifth intervention element is a graphical presentation of progress and success in following the advice, designed to improve the patient’s adherence to HF management. The last type of intervention component is education about HF disease and its treatment, which is provided in a library to be consulted on a voluntary basis. All these different types of interventions are delivered by notifications in a personalized way adapted to the patient’s psychological profile (based on State Trait Anxiety Inventory [26] and Beck Depression Inventory II [27]).

A detailed overview of these intervention components is given in Table 2.

**Table 2:** Overview of the HeartMan intervention components

Intervention component	Strategy	Description of the intervention
Nutrition	Education on: - knowledge of healthy nutrition - eating behavior	*-* Questionnaire to evaluate knowledge and healthy behavior ○ Baseline and end of the trial *-* Personalized feedback and education is coupled to the questionnaire *-* Possibility to consult questionnaire and education material via the application on a voluntary basis during the trial
Exercise	Endurance exercise	*-* Assessment of physical capacity: cyclo-ergometry (if available) and 6MWT *-* Differentiation based on baseline physical capacity level (low level: cyclo-ergometry < 1 W/kg or 6MWT < 300 m vs. normal level: ≥1 W/kg or 6MWT ≥300 m) *-* Individual exercise program via the application: ○ Frequency: 2–5 times per week ○ Intensity: HR rest + 40–70% HRR or RPE 10–14/20 ○ Time: 10-40 min ○ Type: cycling, walking, steps *-* Individual progress: ○ Increase in frequency or time after a predefined number of weeks (after patient’s approval) ○ Increase in intensity if following criteria are met: • Adherence to exercise therapy > 60% of training sessions • Not overshooting the target heart rate in 80% of the training sessions • Patient’s subjective opinion of exercise intensity: ‘no discomfort’ in 80% of the training sessions • After at least 8 weeks of exercise training
	Resistance exercise	*-* Differentiation based on baseline physical capacity level *-* Individual exercise program via the application: ○ Frequency: 2–3 times per week ○ Intensity: 60–70% 1Repetition maximum ○ Time: 1–2 sets per day ○ Type: dynamic upper and lower limb exercises, with no or light weights *-* Individual progress: ○ Increase in frequency or time after a predefined number of weeks (after patient’s approval) ○ Increase in intensity (from no weights to light weights) after a predefined number of weeks (after patient’s approval)
Medication intake	Weekly pill organizer	*-* Passive pill dispenser, with personalized reminder function in HeartMan application: ○ Guidance to prepare medication once per week ○ Daily personalized notifications to remind the patient to take his medication at the right time *-* Assessment of medication adherence by a weekly question whether the patient has taken the recommended medication or not
Appointments		*-* Personalized reminder function in HeartMan application 1 day before the appointment (only upon the patient’s request)
Mental support	Cognitive behavioral therapy	*-* Patient profiling: adaption of the communication according to the psychological profile (anxious, depressed or low motivation) in order to increase adherence to lifestyle interventions
	Mindfulness exercises	*-* Mindfulness exercises are offered to the patient on a daily basis, adapted to the patient’s profile (anxious, depressed or motivated). *-* Different types of exercises: ○ Listening ○ Focusing ○ Awareness ○ Games
Disease education (illness perception)	Education on: *-* Heart failure disease: causes, symptoms *-* Pharmacological treatment: indications, common side effects *-* Sexual dysfunction and sexual activity	*-* Written education in a depository *-* To consult on a voluntary basis

6MWT, six-minute walking test; HR, heart rate; HRR, heart rate reserve; RPE, rating of perceived exertion

### Intervention component for the caregiver: Web interface

Apart from functionalities focusing on patient’s disease management, HeartMan provides valuable information for formal caregivers. Through a web interface, treating physicians may check adherence and progress of their patient by graphical presentations giving an overview of a certain period of time.

### Alert notifications

Once a patient has received the trial equipment and necessary instructions, the HeartMan app is intended as a stand-alone application used by the patient, without additional support of the HF nurse. Since HF patients need to monitor their weight, heart rate and blood pressure on a regular basis, the HeartMan app will remind them to take these measurements. Unexpected results in these parameters are not transferred to the hospital but the patient will get a notification to contact his treating physician. If predefined exercise requirements concerning heart rate or blood pressure are not met before starting the exercise program or exceed the predefined limits during exercise, a similar notification will appear. A helpdesk for technical questions related to the HeartMan system is available on weekdays from 9 a.m. until 4 p.m. This may strengthen the patient’s role in his own disease management. Table 3 presents the cut-off limits of the alert notifications.

**Table 3:** Alert notifications

Parameter	Device	Frequency, time	Alert notification
Weight	Scale	*- weekly measures at rest* • daily or twice a week (configurable by physician) • before breakfast	≥2 kg weight gain in 3 (or less) days
Systolic BP	BP monitor	*- weekly measures at rest* • twice a week • after breakfast and 10 min of resting *- pre-exercise requirement*	> 180 or < 90 mmHg > 180 or < 90 mmHg
Diastolic BP	BP monitor	*- weekly measures at rest* • twice a week • after breakfast and 10 min of resting	> 100 or < 55 mmHg
Heart rate	HeartMan wristband	*- weekly measures at rest* • twice a week • after breakfast and 10 min of resting *- pre-exercise requirement*	> 120 or < 40 bpm > 120 or < 40 bpm
Respiratory rate	HeartMan wristband	*- weekly measures at rest* • twice a week • after breakfast and 10 min of resting	> 24 or < 10 breaths per min

*BP* blood pressure, *bpm* beats per minute

#### Standard care

The HeartMan intervention is provided on top of standard care which is given to all HF patients, regardless of the randomization process. Standard care consists of optimal medical treatment according to the guidelines [3], and written and oral education on HF disease and its management provided by the HF nurse. Regular visits to the treating physician are scheduled several times per year.

#### End-of-study visit

The intervention is terminated after using the system for three to six months. At this time, all participants from the intervention and control group undergo the end-of-study examination in the hospital that entails the same questionnaires and tests as during the baseline visit.

#### Statistical analysis

The main analysis of primary and secondary endpoints will be based on the Intention-To-Treat principle, i.e. including all patients in the analysis who did not drop out within the first 4 weeks of the trial. In order to assess the clinical effect of the HeartMan intervention on the different outcomes, an additional per-protocol analysis will be performed in patients who adhere to the treatment plan for at least 50%. Based on these results, a dose-response analysis will be conducted to verify the relation between the level of adherence and intervention effects.

Prior to each analysis, distribution of the variables will be checked in order to choose correct statistical tests and identify outliers. In all analyses, *p* values < 0.05 will be considered to be statistically significant.

Effects of the proof-of-concept trial will be assessed by analysing baseline and end-of-investigation data between and among treatment groups. The primary endpoint of the HeartMan project is the self-reported improvement in HRQoL. First, T-tests or its non-parametric variant and chi-square tests will be used to compare the characteristics between the groups. Next, in order to assess the effect of the intervention among the different groups, a repeated measures design with time*group interaction effect will be chosen. A similar statistical approach will be performed for the secondary analyses.

### Handling missing data

A great effort will be made to have complete data on outcome measures and to use all obtained information. For early drop-out within 4 weeks after starting the intervention, novel candidates will be foreseen.

For those who drop out in a later phase, efforts will be made to perform the end-of-study examination and if not feasible, these will be considered as missing data. In the case of missing data, some of the outcome measures (e.g. heart rate or blood pressure) can be retrieved through data from the HeartMan system, using the Last Observation Carried Forward (LOCF) principle.

## Discussion

HeartMan aims to provide appropriate and comprehensive guidance on different domains of disease management in HF, tailored to the patient’s medical and psychological profile. This approach is intended to have a positive impact on HRQoL, which is the primary outcome of this trial.

The focus on HRQoL as a primary outcome instead of targeting hard outcomes such as mortality and hospitalization is one of the innovative approaches in comparison with previous mHealth studies. [8–10] HRQoL has gained more attention lately, often as a secondary outcome [11], although it also becomes more prominent as a primary outcome in recent studies [13].

Results on the effectiveness of mHealth technologies are rather mixed. Plausible reasons may be the focus on a single intervention modality (e.g. education) or a lack of patient’s adherence to technology. In HeartMan, the combination of different intervention modalities, adapted to the individual patient’s physical and psychological condition, is a unique approach, which makes HeartMan stand out from related projects. Another aspect that may increase the likelihood of successful adherence is the involvement of patients in the designing process of HeartMan, making it more adapted to the patient’s daily life.

HeartMan is aimed at evaluating the overall impact of the various active interventions on HRQoL. An analysis of the separate effects of the different intervention components will however not be possible due to the design of the current trial. This may be done in the future with specifically designed trials in case the present trial may succeed. The HeartMan trial will be implemented in two countries, which will allow us to gain more insight into the use of HeartMan across different cultures. However, the minor differences in the recruitment process may create a selection bias which has to be investigated in a post-hoc analysis. This study is a proof-of-concept trial, which may lead to preliminary study results and a limited generalizability. Nevertheless, these results may serve as a basis for larger studies in the future. A barrier that may also affect the generalizability is the exclusion of HF patients with end-stage chronic kidney disease or severe cognitive impairment. The reason for excluding these patients is the physical and cognitive demand of the HeartMan intervention, making participation difficult. Finally, this type of intervention with the use of new technologies including a smartphone may make involvement for elderly patients challenging, but a careful design adapted to the user’s perspective together with home visits and a help desk for technical support should make participation feasible even for this elderly population.

In conclusion, HeartMan is technologically the most innovative HF self-management support system to date. This trial will provide evidence whether modern mHealth technology, when used to its full extent, can improve HRQoL in HF.

### Trial status

The HeartMan trial is actively enrolling participants at the moment of manuscript submission.

## Abbreviations

6-MWT: Six-minute walking test
CPET: Cardiopulmonary exercise testing
DSS: Decision support system
ESC: European Society of Cardiology
HeartMan: Personal Decision Support System For Heart Failure Management
HF: Heart failure
HRQoL: Health-related quality of life
LOCF: Last Observation Carried Forward
mHealth: Mobile health
MMSE: Mini Mental State Examination
NYHA: New York Heart Association
PRO: Patient-reported outcome
